# On granular elasticity

**DOI:** 10.1038/srep09652

**Published:** 2015-05-07

**Authors:** Qicheng Sun, Feng Jin, Guangqian Wang, Shixiong Song, Guohua Zhang

**Affiliations:** 1 State Key Laboratory for Hydroscience and Engineering, Tsinghua University, Beijing, China; 2 Department of Physics, Beijing University of Science and Technology, Beijing, China

## Abstract

Mesoscopic structures form in dense granular materials due to the self-organisation of the constituent particles. These structures have internal structural degrees of freedom in addition to the translational degree of freedom. The resultant granular elasticity, which exhibits intrinsic variations and inevitable relaxation, is a key quantity that accounts for macroscopic solid- or fluid-like properties and the transitions between them. In this work, we propose a potential energy landscape (PEL) with local stable basins and low elastic energy barriers to analyse the nature of granular elasticity. A function for the elastic energy density is proposed for stable states and is further calibrated with ultrasonic measurements. Fluctuations in the elastic energy due to the evolution of internal structures are proposed to describe a so-called configuration temperature *T^c^* as a counterpart of the classical kinetic granular temperature *T^k^* that is attributed to the translational degrees of freedom. The two granular temperatures are chosen as the state variables, and a fundamental equation is established to develop non-equilibrium thermodynamics for granular materials. Due to the relatively low elastic energy barrier in the PEL, granular elasticity relaxes more under common mechanical loadings, and a simple model based on mean-field theory is developed to account for this behaviour.

Dense granular materials are collections of distinct macroscopic particles and are widely encountered in engineering and natural hazards^1^,^2^. Due to their discrete and dissipative nature, particles self-organise into various types of coherent structures, such as vortices, even at small Reynolds numbers^3^,^4^ and force networks^5^, which indicates that these particles have a pronounced short range order but no long-range structural order, as represented by the pair correlation function^6^. Such mesoscopic structures have been a long-standing mystery and cause the unique properties of granular materials that are not present in other materials, such as elasto-plastic granular solids, Herschel-Bulkley granular flows, or combinations of the two. Granular elasticity is a key physical quantity that controls the unique thermodynamic, kinetic and dynamic properties of granular materials. The elastic modulus can be determined by measuring the elastic wave velocity in acoustic experiments^7^,^8^ or by applying an infinitesimal strain (e.g., less than 10^−4^) to measure the effective elastic response via discrete element simulations^9^.

Granular materials exhibit significant fluctuations and uncertainties in their physical quantities. The fluctuations in particle velocities and their importance in granular gases were appreciated by A. Einstein in his studies of Brownian motion in the early 1900s^10^. Because velocity fluctuations nearly vanish in dense systems, the fluctuations in the contact stress or elastic energy become more significant^11^. Several ensemble theories for static states have been proposed to explore the influence of granular configurations on the statistical properties of the free volume or contact stress. This was first studied by Edwards and co-workers by proposing a temperature-like parameter *compactivity* *χ* 12, and measurements of *χ* for binary disc packing were recently reported^13^. Different concepts of granular temperatures have been considered in several contexts^14^,^15^,^16^,^17^,^18^. Moreover, granular elasticity inevitably relaxes into more stable states due to its metastable nature, although the process of relaxation is extremely slow under small mechanical agitations, as has been observed in acoustic measurements^19^. Accordingly, elastic relaxation may be a powerful means of understanding the nature of solid- and fluid-like transitions in granular materials.

Understanding the elasticity of granular materials is one of the oldest and most challenging problems in the theory of matter. It may provide an essential bridge to link grain-scale dynamics to complex macroscopic phenomena and facilitate future efforts in establishing a non-equilibrium thermodynamic theory of granular materials^20^,^21^. In this paper, we report on a preliminary exploration of granular elasticity based on the contact stress distributions in 50 static packings of smooth particles at a constant confining pressure of 10 kPa. We then employ a configuration potential energy landscape (PEL) to illustrate the intrinsic variations of the elastic energy. An elastic energy density function is proposed for local stable states. The elastic and kinetic energies for sheared granular flows is probed, and it is found that for well-jammed systems elastic dominates kinetic energy, and energy fluctuations become primarily elastic in nature. An additional granular temperature, called the configuration temperature *T^c^*, is then proposed to denote the elastic energy fluctuations. To describe the transition between neighbouring stable states in the PEL, we propose a simple model for granular elasticity relaxation that is based on mean-field theory.

## Results

### Contact stress and potential energy density

The packing of cohesionless spheres with a finite value of surface friction *μ* is hyper-static except at the isostatic limits as *μ* = 0 or *μ* → ∞. This means that for a single granular packing, many different sets of force networks exist that satisfy the force balance on each particle^11^. Each set of force network configurations may be characterised by a tensorial form variable Ψ, such as the fabric tensor^9^. Force network ensemble (FNE) theory describes fluctuations in the first invariant of the contact stress by averaging over all of the balanced force networks on a single frozen spatial distribution of particle positions^11^. In this study, we focus on the local pressure distribution. Fifty granular assemblies with a confining pressure of 10 kPa were generated using the discrete element method. Each packing consists of *N* frictionless spheres in a cubic box with periodic boundary conditions with *N* = 10,000. To avoid crystallisation, we use a binary mixture of particles with diameter 6.0 mm and 4.2 mm, i.e., the ratio is 1.4. The material properties are *E* = 69.6 GPa, ν = 0.20 and *μ* = 0.0. The particle density *ρ_p_* = 2,650 kg/m^3^ (see METHODS section for simulation details).

**Figure 1:**
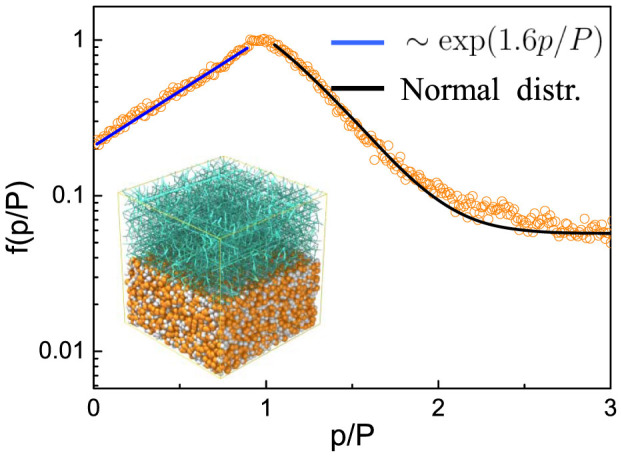
Probability density of the normalised mean pressure p/P. The symbols are calculated from 50 packings with a constant confining pressure of P = 10 kPa. A static granular sample is shown in the inset. The lower part shows particle packings, and the upper part shows the force network.

The inset of Fig. 1 shows the packing (lower part) and force network (upper part) with a structural parameter Ψ. One microscopic parameter is the pressure *p* on an individual grain, where 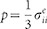 is the trace of the contact stress and is calculated as 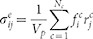, where the summation occurs over all of the contact forces that act on the particle, *N_c_* is the number of contacts of the particle, *V_p_* is the particle volume, 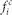 is the *i*-th component of the contact force that acts on the contact, and 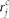 is the *j*-th component of the position vector from the centre particle to the contact. The mean pressure for a granular assembly is expressed as 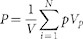, where *V* is the volume of the sample and *N* is the number of particles. In the simulations, *P* is equal to the confining pressure.

Fig. 1 shows the probability density distribution of the normalised pressure *p*/*P*. Similar to the force distribution, a peak is located at approximately *p*/*P* ≈ 1. At the limit of small local pressures *p* < *P*, the local pressure distribution can be fitted as *f*(*p*/*P*) = 0.21 exp(1.6*p*/*P*), and the index value may reflect the local connectivity of the network and the local force balance constraints. In this study, the coordination number of system *Z* was measured as 5.4, and the FNE prediction would be *f*(*p*/*P) ~ (p*/*P*)^1.4^. This difference may arise from the fact that the FNE ensemble is developed from the triangular lattice model, in which the particles are identical and fixed at the lattice positions, whereas the particles in this study are randomly packed. For large local pressures *p* > *P*, *f*(*p*/*P*) exhibits a normal distribution of *f*(*p*/*P*) = 2.20 exp(−(*p*/*P* − 0.19)^2^/0.89^2^)) + 0.057, which is similar to the FNE results. The circumstances in which large pressure distributions are normal or exponential remain the subject of debate^16^.

**Figure 2:**
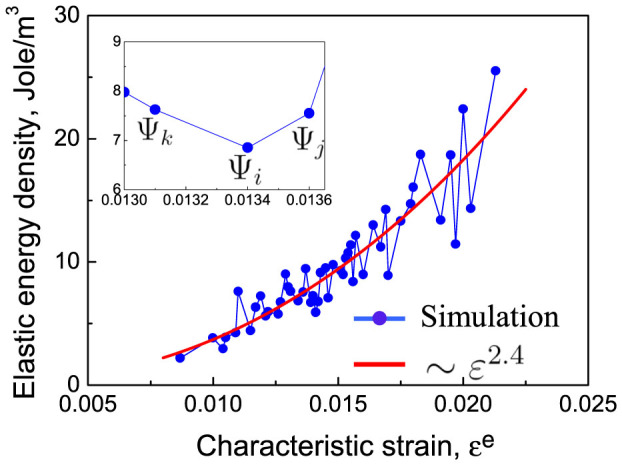
Variations of the elastic energy density with characteristic strain in 50 packings. The enlarged inset shows three pairs of values in packings labelled as Ψ*_i_*, Ψ*_j_*, Ψ*_k_*.

For a given force network Ψ, a characteristic elastic strain at each contact *i* may be defined as 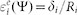, where *δ_i_* is the depth of the contact deformation and *R_i_* is the effective radius of the contacting particles. The elastic potential energy at each contact can be expressed as a power law function of 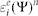; for instance, *n* = 2.5 for Hertzian particles. In a granular assembly, the average potential energy density can be calculated as 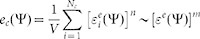, and the average characteristic strain is defined as 
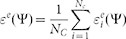
. Fig. 2 implies that at a constant confining pressure of 10 kPa, the characteristic strain *ε^e^*(Ψ) varies from 0.01 to 0.025 due to the variation of the structure variable Ψ in the 50 packings. We can obtain the average elastic potential energy expression by fitting these 50 packings; i.e., *e_c_* = 1.5 × 10^5^(*ε^e^*)^2.4^ J/m^3^, as shown by the red curve in Fig. 2. The fact that both the contact area and strain change simultaneously as the particle is deformed leads to a nonlinear contact response, i.e., *m* is slightly different than *n*.

Under an infinitesimal deformation of a jammed granular assembly, every particle undergoes an extremely slight motion such that the configuration of the force network can completely recover after the loading is removed. Thus, an *elastic* or *reversible* response is defined when the configuration of the force network is unchanged during mechanical deformation in the applied fields. Although extensive work has been performed on granular elasticity using numerical simulations^9^,^22^ or ultrasonic detection^8^, there is no general expression for granular elasticity. A Green elastic density function may be appropriate for an ideally elastic stage, such as 
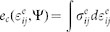
. The elastic moduli are directly dominated by the second derivative of the potential energy that corresponds to the current force network state Ψ.

In granular solid hydrodynamics (GSH)^23^,^24^, an elastic energy density function is defined as 
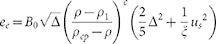
 where *ρ* is the mass density of the system. *ρ_lp_* and *ρ_cp_* are the random loose packing density and close packing density^15^, respectively. 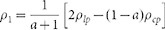. 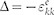 and 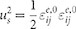 are the first and second invariants of the contact strain 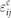 of the particles, respectively, and 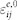 is the traceless part. As a granular system enters the well-jammed state (i.e. *ρ* → *ρ_cp_*) the calculated elastic energy and elastic stress would both become infinitely large, *e_e_* → ∞, 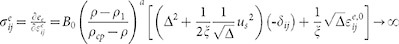. The fact is that the elastic energy density of a granular material should not exceed the elastic energy density of constituent particles. Consequently, untrue elastic energy and stress, and thus bulk properties of granular materials in solid-like states may be incorrectly predicted.

Recent jamming studies find that mechanical properties of granular material after jammed can be well scaled with the distance to Point *J*, i.e (*ϕ* − *ϕ_c_*) 25,26. In this study, we are inspired to revise the elastic energy density as
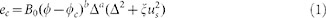
where *B*_0_ is an elastic constant, (*ϕ* − *ϕ_c_*) reflects the influence of the packing fraction after the system is jammed with the critical packing fraction *ϕ_c_* (c.f. 25,27). The parameters 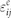 and *ϕ* are independent from each other, and both contribute to the macroscopic strain. In studies of the micro-macro quantification of the internal structure, particles are often assumed to be rigid bodies, and thus, the deformations of individual particles are always neglected. In this work, both 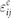 and *ρ* (i.e., *ϕρ_p_* with a particle density *ρ_p_*) are chosen as state variables to develop the thermodynamic descriptions in the next section, whereas in jammed systems (*ϕ* > *ϕ_c_*), particles overlap, a contact strain 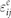 is generated, and the invariant Δ, *μ_s_* should be related to (*ϕ* − *ϕ_c_*); however, the relationships are not known. In Equation (1), the four factors *B_0_*, *a*, *b* and *ξ* are functions of both the structural variable Ψ and the material properties of the particles, such as the modulus, size distribution, and friction^28^,^29^,^30^.

**Figure 3:**
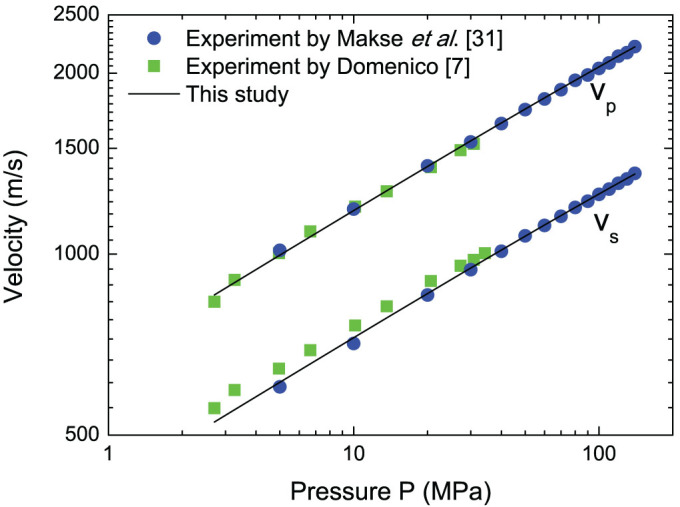
Wave velocities versus pressure obtained in this study and from previous experiments^7^,^31^.

The acoustic method can ensure that accurate parameters are used in the elastic potential, which provides us with an important way to study the nonlinear elasticity behaviour of granular materials in detail if the stresses, densities, and uniformity of the sample are thoroughly controlled and measured. In isotropic compression, the static pressure *P* in a granular assembly can be derived as
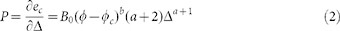
and the longitudinal wave velocity *v_p_* and transversal wave velocity *v_s_* are
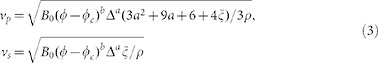
In the acoustic experiments by Makse et al.^31^, glass beads were used. The particle diameter of was 45 μm. The typical material properties were *E* = 69.6 GPa, ν = 0.20 and *μ* = 0.0. The particle density *ρ_p_* = 2,650 kg/m^3^. They found that 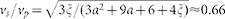 in isotropic compression tests. The contact potential of cohensionless spherical glass beads can be reasonably treated as Hertzian potential; thus, we simply set *a* = 0.50 in this study, and obtain *ξ* = 3.88. In their experiment, *ϕ_c_* was measured as 0.63. In this study, by employing the numerical technique to determine *ϕ_c_* 32, *ϕ_c_* ≈ 0.635 is determined for 10,000 uniform glass beads under isotropic compression. The factors *B*_0_ = 9.22 × 10^9^ Pa, *b* = 0.39 are obtained by comparing wave velocities calculated in this study and from previous experiments in Ref. 31, as shown in Fig. 3. It demonstrates that acoustic methods are appropriate to determine the factors in the revised elastic energy density function in future applications in specific granular materials.

### Elastic energy fluctuations and configurational temperature

Most thermodynamic systems at equilibrium states have strong scale separation, and the concept of temperature may be an important characterisation of fluctuations in the molecular velocity. In statistical mechanics, the second moment of the internal energy fluctuations is related to temperature as
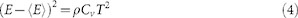
where *C_v_* is the thermal capacity of the system. In heterogonous systems, the energy is not equally distributed among the several degrees of freedom, and different degrees of freedom with different relaxation times may have different temperatures^33^,^34^. A classic example of such a system is found in plasma physics. Here, electrons and ions may have widely different kinetic energies, and the energy exchange through collisions is extremely slow. This situation may be described by the equations for the evolution of the electron and ion temperatures, respectively.

**Figure 4:**
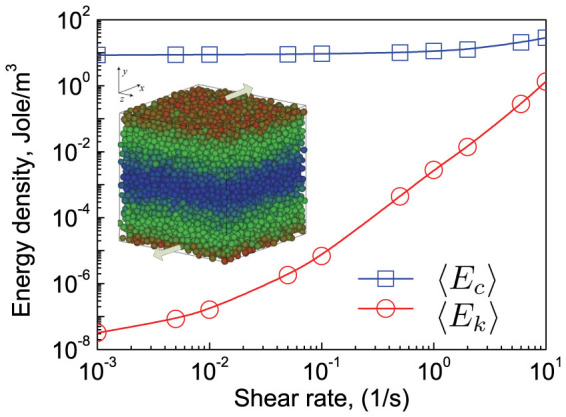
Variations of the mean kinetic energy (circles) and mean elastic energy (squares) with shear rates. The inset shows an example of shear velocity distribution. A darker shading indicates slower velocities.

**Figure 5:**
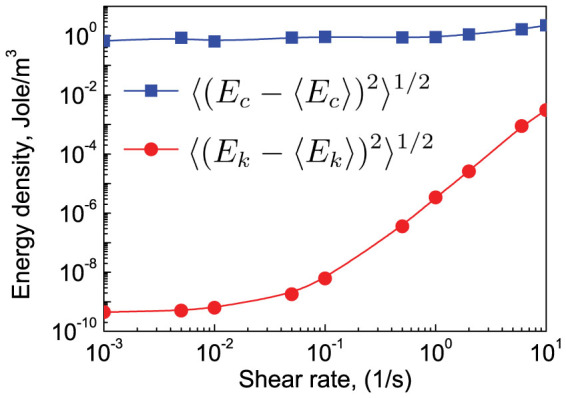
Variations of the fluctuated kinetic energy (circles) and fluctuated elastic energy (squares) with shear rates.

We analyse the mean and fluctuating parts of the elastic and kinetic energy of a jammed granular flow under simple shear. The material properties are *E* = 69.6 GPa, ν = 0.20 and *μ* = 0.2. The particle density *ρ_p_* = 2,650 kg/m^3^. *α* = 0.07, corresponding to a typical restitution coefficient of 0.9. The volume (i.e. *ϕ* = 0.648) is constant. To avoid crystallisation, we use a binary mixture of particles with diameter 6.0 mm and 4.2 mm. The steady state velocity distribution in the *x*-direction for a typical case is shown in Fig. 4. Darker shading indicates slower velocities. Besides energy dissipation, the system's energies are the elastic potential energy and the kinetic energy stored in particles. The kinetic energy *E_k_* is a summation over the kinetic energy of all the particles. The elastic energy is a summation over the elastic energy of all contacts, 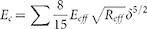 where *δ* is the inter-particle overlap. The mean parts of the elastic and kinetic energy are 〈*E_k_*〉 and 〈*E_c_*〉 shown in Fig. 4. 〈*E_c_*〉 slightly increases from 8.70 Jole/m^3^ to 54.20 Jole/m^3^, while 〈*E_k_*〉 increases nearly 8 orders. 〈*E_k_*〉 is much smaller than 〈*E_c_*〉. Even at shear rate of 10.0/s, 〈*E_c_*〉 is still about 25.5 times of 〈*E_k_*〉. According to equation (4), the fluctuating parts of the elastic and kinetic energy are defined as 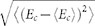 and 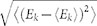, respectively. The results are shown in Fig. 5. It is found that 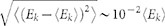 and 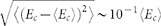. For the whole range of shear rates, 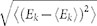 is smaller by at least 3 orders of magnitude than 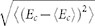. It clearly shows that for well-jammed systems elastic dominates kinetic energy, and energy fluctuations become primarily elastic in nature.

The *kinetic granular temperature* *T^k^* measures the fluctuations in the translational velocity of the particles and can be defined as 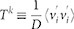, where *D* is the dimension of the system, 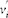 is the fluctuation in the *i-*th component of the velocity of a particle, and 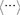 indicates the ensemble average^35^,^36^,^37^,^38^,^39^. Several remarkable properties of *T^k^* have been reported, including the decay of the temperature in granular gas (i.e., the so-called Haff's law), non-homogeneous cluster formation, and shock wave propagation^39^,^40^,^41^. However, non-equilibrium statistical mechanics are still not well developed for discussions of the kinetic granular entropy *S^k^* (the conjugate variable of *T^k^*), the transport coefficients and the entropy production rate^42^. The ensemble-averaged kinetic energy fluctuation should be rigorously expressed as 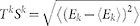; however, in most textbooks, it is simply written as *ρT^k^*. Figs. 4 and 5 clearly demonstrate *T^k^* does not suffice to even approximately describe a dense slowly sheared granular system, and it is essential to incorporate elastic energy and its fluctuations.

By considering the translational and internal structural degrees of freedom in granular materials, a two-granular-temperature description is appropriate to apply for the two different degrees of freedom. As a granular assembly is jammed, a force network is established, and an additional parameter that indicates the structural state of the material is required. We introduce the *configurational granular temperature* *T^c^* to account for fluctuations in the contact stress in the force network. The ensemble-averaged elastic potential energy fluctuation can be expressed as *E^c^*^,*f*^ = *T^c^S^c^* with the configurational granular entropy *S^c^*. The determination of *S^c^* is one of the main themes in studies of the statistical mechanics of static granular materials. The rigorous definition of *T^c^* would be rather complicated due to the tensorial nature of elastic stress and strain. At the preliminary stage, *T^c^* may be simply defined as *T^c^* = 〈*p*′*p*′〉^1/2^, where *p*′ = *p* − *P* is the fluctuation pressure (see Fig. 1). The average elastic energy fluctuation may be simply expressed as 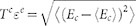. This consideration of *T^c^* is not perfect, but might view this as a further step from GSH towards a more reasonable thermodynamics description of dense granular systems. The ultimate test will be to perform physical experiments where the validity of the proposed concept of *T^c^* can be verified.

At the primary stage, the absolute zeros of *T^k^* and *T^c^* can be examined. *T^k^* = 0 indicates that all particle velocities are the exactly the same as the velocity of the frame. For orderly packed identical particles under isotropic loading, the pressure on each particle is the same such that *T^c^* is zero. *T^c^* also vanishes in unjammed systems because there is no force network. Large values of *T^c^* indicate large divergences in the particle stress, which we observed as strong force chains and weak force chains in photo-elastic tests. Because of the mutual influences of the internal processes, energy would transfer between several degrees of freedom. *T^c^* may partially transfer to *T^k^*, which is often observed as stress stick-slip and simultaneous rearrangement of local particles. Both *T^k^* and *T^c^* would dissipate rapidly into heat, but heat cannot easily transfer to *T^k^* and *T^c^*.

### A two-granular temperature thermodynamics fundamental equation

Additional variables that indicate the structural state of matter typically need to be incorporated into the development of a reliable thermodynamic connection between fundamental micro-mechanical models and continuum-level models. In granular materials, by keeping the basic variables in the equilibrium state, the space of state variables are enlarged with the two non-equilibrium variables, namely, the kinetic granular entropy density *s^k^* and configurational granular entropy density *s^c^*:
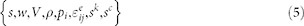
The thermodynamic quantities are all relative to a unit volume of the deformed system. The subscripts *i, j*, and *k* indicate the space coordinates in the Cartesian coordination system and satisfy the Einstein summation convention. The conjugate variables of the energy density *w* are temperature *T* = *∂w*/*∂s*, chemical potential *μ* = *∂w*/*∂ρ*, velocity *v_i_* = *∂w*/*∂p_i_* = *ρv_i_*, contact stress 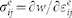, and two granular temperatures *T^k^*_ = _*∂w*/*∂s^k^* and *T^c^*_ = _*∂w*/*∂s^c^*. The validity of the concepts of *T^k^* and *T^c^* should be investigated carefully because the chaotic assumption may not hold further. However, the values of *T^k^* and *T^c^* can still be used to qualitatively describe the degree of shearing or perturbation. We assume that the process of deformation occurs so slowly that thermodynamic equilibrium is established in the granular systems at every instant according to the external conditions.

The fundamental thermodynamic equation would be
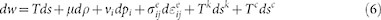
where (*Tds* + *μdρ*) is the internal energy that is contributed from the microscale, *v_i_dp_i_* is the mean kinetic energy, *T^k^ds^k^* is the fluctuation kinetic energy, 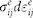 is the mean elastic energy, and *T^c^ds^c^* is the fluctuation elastic energy. The thermodynamic pressure *P* of granular flows is generated only by the random motions of the particles *P* = *Ts* − *w* + *p_i_v_i_* + *μρ* + *T^k^s^k^* + *T^c^s^c^*. For relatively simple cases, the fundamental thermodynamic equation (6) may be simplified as 1) *dw* = *Tds* + *μdρ* + *v_i_dp_i_* + *T^k^ds^k^* and 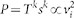 for granular gases^34^,^35^,^36^,^37^,^38^,^39^,^40^,^41^, and 2) 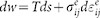 and *P* = 0 for quasi-static deformations of granular solids. A similar expression for the fundamental equation for ordinary elastic solids was derived by Landau and Lifshitz^43^.

The equations for the evolution of the hydrodynamic variables (e.g., mass, momentum, energy and entropy) are given by the normal balance laws. 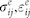 can be determined once the elastic energy (Equation (1)) is specified. However, no general criteria for the equations for the evolution of granular temperatures or granular entropies exist, with the exception of the restrictions imposed on them by the second law of thermodynamics. Specifying the time evolutions of *T^k^* and *T^c^* and quantifying energy cascading, especially the entropy production rates, would be crucial points in future studies of the thermodynamic descriptions of granular materials. A preliminary scenario of energy cascading was proposed as a two-stage irreversibility, i.e., the transitions from hydrodynamic variables to granular temperature and then to heat^23^.

Moreover, a generalisation of the kinetic temperature and configurational temperature would be more feasible than employing two granular temperatures. A unified granular temperature *T^g^* can be defined as *T^g^ds^g^* = *T^k^ds^k^* + *T^c^ds^c^*, which includes the collective fluctuations of both the kinetic and elastic potential energy. The fundamental equation (6) would then be simplified to 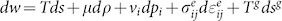. This is the fundamental equation in so-called granular solid hydrodynamics, in which a single granular temperature was used to quantify the extent of agitation^24^.

### Elasticity relaxation

**Figure 6:**
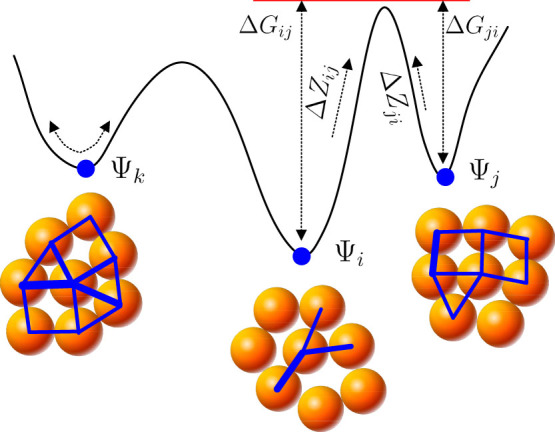
Schematic illustration of a PEL that arises from the disordered nature of the force network and its inherent fluctuations. The bottom of each basin corresponds to a given realisation of the force network. The three packings Ψ*_i_*, Ψ*_j_*, Ψ*_k_* are calculated in Fig. 2. The energy barriers between the basins are depicted schematically. The barrier-crossing event leads to a constrained configuration change of the force networks.

Classical thermodynamics typically interprets the slow relaxation of protein folding and glasses in terms of a PEL with simple structural features^44^,^45^. The PEL is an analogue of the topological surface but in a multi-dimensional configuration space. For granular materials, the quantities can be preferably chosen as elastic energy (*e_c_*)-related variables, such as the force network structure variable Ψ proposed in this work. We can clearly illustrate the correlations between Ψ and *e_c_* on the PEL, which is shown schematically in Fig. 6. The landscape of the PEL is intrinsically rough with basins that are separated by energy barriers. Three blue points with the structural variables Ψ*_i_*, Ψ*_j_* and Ψ*_k_* and their elastic energy densities are also shown in Fig. 2. The energy barriers are depicted schematically in this study, and the volume of the potential energy basins can be measured using the Monte Carlo method^46^. At each basin bottom the potential energy is of local minimum and corresponds to a given realisation of the force network. It would be stable if the mechanical excitation is smaller than the energy barrier; see point Ψ*_k_*. In this case, Ψ*_k_* is reversible upon unloading, and high-frequency relaxations would correspond to mechanical excitations of the state. The elastic moduli are directly dominated by the second derivative of the potential energy in the structural state Ψ*_k_*. Equation (1) for the potential energy would hold through a succession of deformations at each basin in the PEL as a function of the corresponding Ψ.

Acoustic tests have shown that the elastic modulus of a granular material is generally 10^3^ times smaller than that of the constituent particles due to structural disorder^8^. For example, the modulus of quartz is on the order of tens of GPa, whereas the modulus of a sand pile is on the order of MPa. Thus, elasticity relaxation, which is essentially a rearrangement of force networks, would inevitably occur under typical mechanical loadings and play an important role in changing the elastic modulus of granular materials (see the transition between Ψ*_i_* and Ψ*_j_* in Fig. 6). Such transitions between neighbouring basins represent slow dynamic relaxation. As Ψ evolves, the point that represents the system travels on the PEL while following certain ensemble statistics (c.f.11,12). The number of available basin minima at the corresponding energy level on the PEL may be quantified with the configuration entropy *S^c^*. The packing structure, potential energy, configuration entropy and effective energy barrier of a basin all correlate with one another based on the PEL interpretation presented above.

Inspired by the analysis of an elastic deformation in metallic glass^47^, we develop a simple mean-field model to quantitatively determine the elastic relaxation in granular materials using transition state theory. Under mechanical agitation that is quantified with the unified granular temperature *T^g^*, the transition rates 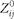 and 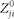 between two adjacent stable states *i* and *j* can be expressed as 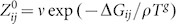 and 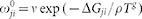, respectively, where *v* is the attempt frequency and Δ*G_ij_* and Δ*G_ij_* denote the two energy barriers illustrated in the inset of Fig. 6. In equilibrium states, 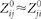 is a first-order approximation such that any spontaneous flow of transitions can be neglected over short periods. If we assume that the energy potential is biased toward the stable state *j* upon a mechanical perturbation 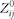 of the system, then we can approximate the new transition rates by 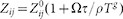 and 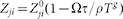, where *τ* and Ω denote the applied shear stress and activation volume of the force network, respectively, and satisfy Ωτ/*ρT^g^*_ ≪ 1_. Assuming that *N_i_* and *N_j_* constrained structural transitions occur and transform many of the force networks from the stable state *i* to *j* and back to *i*, we can write
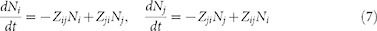


The net flow of transitions in the loading direction is governed by
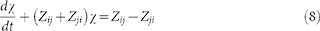
where *χ* = (*M* − *N_i_*)/*M* = (*N_j_* − *M*)/*M*, in which *M* = (*N_i_* + *N_j_*)/2 remains a constant throughout the process. Substituting the expressions for *Z_ij_* and *Z_ji_* into this equation yields
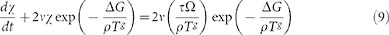
with Δ*G* = Δ*G_ij_* ≈ Δ*G_ji_*.

We can postulate how the generalised temperature *T^g^* determines which level of the PEL is being sampled. If a system is highly excited, the energy fluctuation (*T^g^*) would be so high that exp(−Δ*G*/*ρT^g^*) ~ 1; granular elasticity is lost completely, which means that the material enters the gaseous state. The system would travel globally around the PEL and exhibit ergodicity. When *T^g^* is sufficiently low that Δ*G* ≫ *ρT^g^*, such activation events are greatly suppressed (i.e., exp(−Δ*G*/*ρT^g^*) ~ 0). The PEL becomes increasingly hierarchical, and the elastic relaxation bifurcates into basin hopping. This model provides an analytical framework to explain several phenomena that have been recently explored and supports the theory that the variability in the local rheological properties of granular materials is due to the intrinsic structural heterogeneity.

## Discussion

Granular elasticity originates from enduring interparticle contacts, whereas the emergent force network structures generate a greater variety of stable states, significant fluctuations and clear relaxations than in molecular systems. The PEL employed in this work provides a complete scenario of granular elasticity. The proposed elastic energy density function can be calibrated simply with acoustic experiments and is consistent with the major conclusions obtained in recent jamming studies. The configuration temperature *T^c^* in this study is an extension of the kinetic temperature *T^k^* that has been used in most granular kinetics since the 1980s. Both temperatures would be valid for a wide range of regimes of granular materials from static to dynamic and from dilute to dense. The elastic relaxation model is based on mean-field theory and neglects the interactions between different structures when the stress is lower than a certain value. This work was developed specifically for granular elasticity and emphasises the mesoscopic structure and macroscopic elasticity relationships. Furthermore, it may suggest a general methodology for handling a wide range of existing complex systems and their dynamic heterogeneity.

## Methods

### Discrete element simulations

The contact model we use follows Hertz-Mindlin contact theory with Coulomb sliding friction^48^. The contact forces in the directions normal and tangential to the contact plane between two contacting particles, *F_n_* and *F_t_*, are expressed as
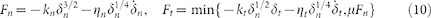
where *δ_n_*, *δ_t_* are the corresponding deformations and *μ* is the coefficient of friction. The coefficients can be calculated as 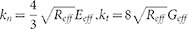, 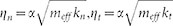
where 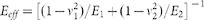, *G_eff_* = [2(1 + *v*_1_)(2 − *v*_1_)/*E*_1_ + 2(1 + *v*_2_)(2 − *v*_2_)/*E*_2_]^−1^ and *R_eff_* = [1/*r*_1_ + 1/*r*_2_]^−1^. Subscripts 1 and 2 refer to the two particles that are in contact, and *E_i_*, *v_i_*, *m_i_* and *R_i_* are the Young's modulus, Poisson's ratio, mass and radius of particle *i*, respectively. To prepare a static packing at a constant confining pressure, 10,000 particles are initially generated in a cubic box, and the box then shrinks to ensure that the packing fraction *ϕ* increased by steps of 1 × 10^−5^. After each increment step of *ϕ*, the particles are allowed to relax for a sufficiently long period of time to allow the system to reach a static state. The stopping criteria for each step were 1) the mean stress *P* for successive iterations deviated by less than 10^−5^ Pa, and 2) the mean stress remained at a constant value of *P* = 10 kPa.

To simulate a simple shear flow, we follow the standard techniques developed for non-equilibrium molecular dynamics. The velocity in the *x*-direction *v_x_* has a constant gradient in the *y*-direction, as shown in Fig. 4. When a particle moves out of the computational domain in any direction, it re-enters from the opposite direction. The domain is expanded or compressed to the desired volume according to the prescribed packing fraction. All data are obtained after the shearing motion has reached a steady state, as detected by observing the time series of the stresses. The granular system is sheared using a shear rate of *v_x_*/*L* with length *L*.

### Elastic wave velocity analysis

To investigate the propagation of elastic waves in granular materials, one can use the general equation of motion 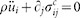 and the contact stress 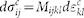, where *u_i_* is the elastic displacement vector in elastic waves, and 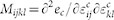 is the stress-dependent stiffness tensor which can be derived from Equation (1),
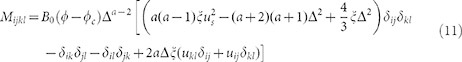


Assuming 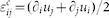 and 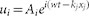, the equation of motion is re-written as
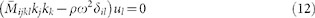
with 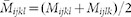 with the stiffness tensor. They have non-zero solutions only if the determinate of the coefficient is zero, i.e. 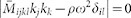. Substituting *k*^2^ = *k_i_k_i_*, *n_i_* = *k_i_*/*k* and the wave velocity *v* = *ω*/*k* yields
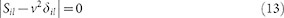
with the wave tensor 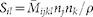 and the eigenvalue *v*^2^. In the case of propagation along the principal stress axes *S_il_* becomes diagonal.

Assuming wave propagation along the axe of 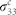, **n** = (0, 0, 1), three wave velocities *v_i_* and corresponding displacement vectors **u***_i_* can be expressed as
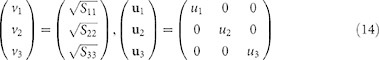
where *S*_11_ = (*M*_1331 _+ *M*_1313_)/2, *S*_22_ = (*M*_2332 _+ *M*_2323_)/2. We can see that the elastic waves traveling along direction **n** are either pure transversal (**u**_1_, **u**_2_) or longitudinal (**u**_3_) modes, i.e. *v*_1_ and *v*_2_ are the transversal wave velocity, and *v*_3_ is the longitudinal wave velocity. For an isotropically confined granular assembly, the stiffness tensor component would be *M*_1331 _+ *M*_2323_, *M*_1331 _+ *M*_2323_. From Equation (1, 11), we obtain 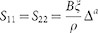 and 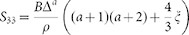. Hence, the transversal wave velocity and longitudinal wave velocity are 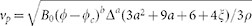 and 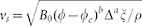.

## Author Contributions

Q.S. proposed and supervised the project. F.J. and G.W. advised on the project. S.S. and G.Z. carried out the elastic wave velocity analysis and discrete element simulations. Finally, the manuscript was prepared by Q.S., S.S. and G.Z. and revised by F.J. and G.W.
